# Medicinal mushrooms and type 2 diabetes: mechanistic insights, translational evidence, and clinical caveats

**DOI:** 10.3389/fnut.2026.1906859

**Published:** 2026-09-14

**Authors:** Radoslav Z. Pavlović, Jovana N. Novaković, Galina V. Ramenskaya, Aleksandar N. Luferov, Vladimir Lj Jakovljević, Vladimir S. Kurćubić, Nevena S. Jeremić

**Affiliations:** 1 Department of Chemistry, Institute of Chemistry, Technology and Metallurgy, University of Belgrade, Belgrade, Serbia; 2 Department of Pharmacy, Faculty of Medical Sciences, University of Kragujevac, Kragujevac, Serbia; 3 Center of Excellence for the Study of Redox Balance in Cardiovascular and Metabolic Disorders, Faculty of Medical Sciences, University of Kragujevac, Kragujevac, Serbia; 4 Sechenov First Moscow State Medical University (Sechenov University), Moscow, Russia; 5 Department of Physiology, Faculty of Medical Sciences, University of Kragujevac, Kragujevac, Serbia; 6 Department of Human Pathology, I.M. Sechenov First Moscow State Medical University (Sechenov University), Moscow, Russia; 7 Department of Food Technology, Faculty of Agronomy, University of Kragujevac, Čačak, Serbia

**Keywords:** functional foods, fungal bioactives, fungal fibers, gut microbiota, insulin resistance, mushroom polysaccharides, oxidative stress, β-glucans

## Abstract

Type 2 diabetes mellitus (T2DM) is a highly prevalent and rapidly expanding global metabolic disorder. Medicinal mushrooms have recently been proposed as promising functional foods, owing to their broad range of bioactive compounds, including β-glucans and other polysaccharides, triterpenoids, phenolic metabolites, sterols, nucleoside analogs, and sulfur-containing antioxidants. Their bioactivity is increasingly understood as an emergent property of multi-scale interactions, in which structural glycan complexity, supramolecular organization, and chemically diverse low-molecular-weight metabolites act in a network-like fashion rather than through single-target mechanisms. This review provides an integrated synthesis of the antidiabetic potential of medicinal mushrooms, encompassing ethnopharmacological origins, chemical diversity, preclinical and clinical evidence, and mechanistic pathways. Preclinical studies consistently report improvements in glucose homeostasis, insulin sensitivity, lipid metabolism, and redox balance across diverse experimental models. These effects are associated with modulation of insulin–PI3K/Akt and AMPK signaling, Nrf2-dependent antioxidant responses, NF-κB–driven inflammatory pathways, and gut microbiota–host metabolic interactions involving short-chain fatty acids and incretin signaling. However, most mechanistic evidence remains pathway-associated rather than causally validated, and the clinical relevance of many findings is limited by uncertain dose–response relationships, sparse pharmacokinetic data, preparation heterogeneity, and incomplete safety evaluation. Mushroom–drug interactions, liver safety, and environmental contaminant screening of concentrated mushroom-derived products warrant particular attention. Together, these factors constrain clinical translation, further compounded by heterogeneity of interventions, lack of standardization, short trial durations, and limited integration of mechanistic endpoints. Overall, medicinal mushrooms show potential as multi-target functional foods capable of modulating core pathological networks in T2DM. Future progress will critically depend on rigorous standardization of bioactive preparations, mechanistically driven clinical trials, and systems-level multi-omics integration to establish causal links between fungal bioactives and metabolic outcomes.

## 1 Introduction

Type 2 diabetes mellitus (T2DM) is one of the most pressing contemporary health challenges. It is the fastest growing chronic metabolic disorder worldwide linked to obesity, sedentary lifestyle, aging, and unhealthy dietary patterns. Recent global estimates indicate that approximately 828 million adults were living with diabetes in 2022, representing an increase of more than 600 million cases since 1990, with the burden increasingly concentrated in low- and middle-income countries (1). Beyond its devastating impact on human health, T2DM imposes an enormous socioeconomic burden, with global diabetes-related healthcare expenditures estimated to exceed hundreds of billions of U.S. dollars annually and projected to rise substantially in the coming decades (2). As evident from Figure 1A, T2DM is an extremely active area of research driven by the urgent need to develop effective preventive, nutritional, and therapeutic strategies to address its growing global health and socioeconomic burden.

**FIGURE 1:**
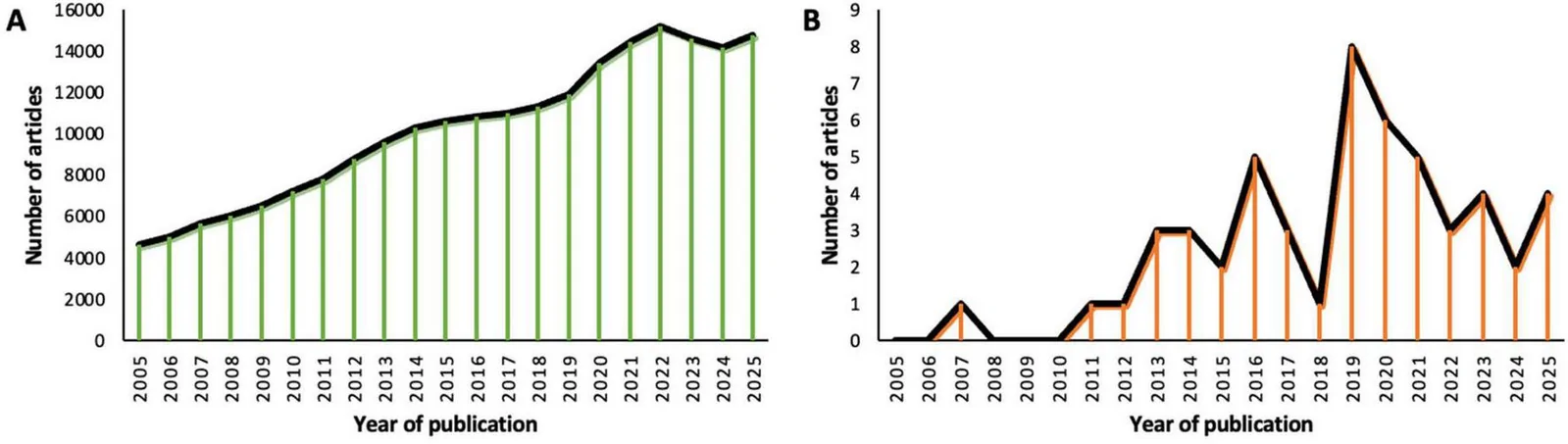
Growth of scientific interest in T2DM and in medicinal mushrooms for its management between 2005 and 2025. **(A)** Annual number of PubMed-indexed publications related to T2DM. **(B)** Annual number of PubMed-indexed publications focusing on medicinal mushrooms in T2DM. The parallel upward trends illustrate the rapidly increasing scientific interest in both T2DM research and the therapeutic potential of medicinal mushrooms.

The pathophysiology of T2DM involves a progressive disruption of glucose homeostasis driven by insulin resistance (IR), impaired pancreatic β-cell compensation, chronic low-grade inflammation, oxidative stress, and dysregulated lipid metabolism, accompanied by altered crosstalk among adipose tissue, liver, skeletal muscle, and the immune system (3, 4). Oxidative stress is tightly coupled with inflammatory signaling pathways, including nuclear factor kappa B (NF-κB), further exacerbating insulin resistance and metabolic imbalance (5). Conversely, activation of endogenous antioxidant defense systems, most notably those mediated by nuclear factor erythroid 2-related factor 2 (Nrf2), has been shown to improve insulin sensitivity and preserve metabolic homeostasis (6). Together, these convergent processes establish a self-perpetuating cycle of metabolic and immune dysregulation that contributes to persistent hyperglycemia. As the disease progresses, chronic hyperglycemia contributes to microvascular and macrovascular complications and increases overall mortality.

Despite the availability of pharmacological therapies, the global prevalence of T2DM continues to rise, emphasizing the urgent need for preventive strategies centered on nutrition, dietary modulation, and lifestyle intervention (7). Importantly, the pathophysiological continuum from normoglycemia to T2DM typically includes a prolonged prediabetic stage, during which metabolic dysfunction is already present but remains potentially reversible. Early intervention at this stage is critical, as lifestyle and dietary strategies have been shown to significantly reduce the risk of progression to overt diabetes (7–9).

Diet and lifestyle are major modifiable determinants of T2DM risk. Accordingly, nutritional approaches are central to preventive nutrition research and clinical practice. Functional foods and naturally occurring bioactive compounds are especially attractive because they may influence multiple metabolic pathways while maintaining favorable safety profiles (10). According to the European consensus definition, a food may be considered functional if it beneficially affects one or more target functions beyond adequate nutrition in a manner relevant to improved health, well-being, or reduced disease risk, while remaining part of a normal dietary pattern rather than being consumed as a pill or capsule (11). This concept is highly relevant to T2DM prevention, where dietary components may modulate postprandial glycemia, insulin sensitivity, oxidative stress, inflammation, lipid metabolism, gut microbiota composition, and body weight.

Unlike conventional single-target pharmacological approaches, bioactive compounds from mushrooms may influence several integrated processes involved in T2DM, including glucose and lipid metabolism, insulin signaling, oxidative stress, inflammatory responses, gut microbiota composition, immune regulation, and β-cell function. Several edible and medicinal mushroom species including *Ganoderma lucidum*, *Inonotus obliquus*, *Pleurotus ostreatus*, *Grifola frondosa*, *Lentinula edodes*, *Cordyceps* spp., and *Hericium erinaceus*, are rich in polysaccharides, β-glucans, terpenoids, sterols, phenolics, peptides, and antioxidant metabolites. These constituents have potential relevance to insulin resistance and chronic metabolic dysfunction. This rising scientific interest is reflected in the steady increase in publications at the intersection of medicinal mushrooms and diabetes over the past two decades (Figure 1B). Although human evidence remains limited and inconsistent, a prospective Korean cohort study reported an inverse association between mushroom consumption and T2DM incidence in adults aged over 40 years (12), and systematic reviews of clinical trials suggest possible benefits on fasting and postprandial glucose and lipid parameters, most consistently for *Pleurotus ostreatus*, while noting that the overall evidence remains limited and heterogeneous (13, 14).

Collectively, the available evidence positions medicinal mushrooms as promising candidates for integrative nutritional strategies aimed at improving metabolic resilience and reducing long-term cardiometabolic risk associated with T2DM.

Accordingly, this review examines medicinal mushrooms and fungal bioactives as emerging functional-food candidates for the management of established T2DM. Particular emphasis is placed on their ethnopharmacological background, chemical diversity, preclinical and clinical evidence, and the molecular mechanisms underlying metabolic, redox, immunological, and gut microbiota-mediated regulation.

## 2 Ethnopharmacological roots of medicinal mushrooms as functional foods

Medicinal mushrooms occupy a distinctive position at the intersection of traditional medicine, nutrition, and modern functional-food science. Their ethnopharmacological relevance reflects a long-standing historical relationship between humans and fungi, particularly within Asian, Mesoamerican, Eastern European, and Russian medical traditions (15). For centuries to millennia, species such as *Ganoderma lucidum*, *Inonotus obliquus*, *Fomitopsis officinalis*, and termite-associated mushrooms of the genus *Termitomyces* have been used in traditional practices for vitality, gastrointestinal disorders, cardiovascular complaints, and metabolic conditions (16, 17). Although historical use does not substitute for clinical evidence, ethnopharmacological knowledge provides an important discovery framework for identifying fungal species and metabolites with potential relevance to T2DM prevention and management.

Modern studies increasingly support this rationale, identifying polysaccharides, β-glucans, terpenoids, sterols, phenolics, proteins, dietary fibers, and other fungal metabolites capable of modulating glycemic control, insulin sensitivity, oxidative stress, inflammation, gut microbiota composition, and β-cell function (18, 19). This broad spectrum of bioactive constituents is thought to underlie the anti-inflammatory, antioxidant, immunomodulatory, and antidiabetic properties attributed to medicinal mushrooms. However, despite extensive historical use and growing experimental evidence, standardization of mushroom-derived bioactives remains a major challenge due to substantial variability in species, cultivation conditions, extraction procedures, and chemical composition (20, 21).

Given the growing global burden of T2DM and the limitations of current therapeutic strategies, increasing attention has been directed toward complementary nutritional and functional-food approaches capable of modulating multiple metabolic pathways simultaneously (22). Within this context, edible and medicinal mushrooms have emerged as promising sources of antidiabetic bioactives owing to their chemically diverse molecular composition and reported effects on glycemic regulation and metabolic homeostasis (23). The functional-food relevance of mushrooms is further supported by their nutritional profile (24): they are generally low in calories, fat, and sodium, while being rich in high-quality proteins, dietary fibers, vitamins (particularly B and D vitamins), and essential minerals (25, 26). Together with their diverse repertoire of secondary metabolites, these nutritional characteristics position medicinal mushrooms as attractive candidates for integrative dietary strategies targeting metabolic disorders. As interest in preventive nutrition and metabolic health continues to expand, medicinal mushrooms are increasingly transitioning from traditional ethnomedicinal remedies toward evidence-based functional-food candidates with potential relevance for long-term cardiometabolic health management (26, 27).

## 3 Bioactive compounds from medicinal mushrooms relevant to T2DM

Glycans are the most abundant and structurally diverse class of biomolecules on Earth, yet their analytical complexity has historically slowed progress in glycoscience relative to other major molecular classes (28–31). Beyond serving as energy reservoirs, glycans participate in a broad spectrum of biological processes, including cellular communication, signal transduction, immune regulation, molecular recognition, and cell adhesion (30, 32). Through their structural diversity and dynamic presentation within biological environments, glycans play fundamental roles in governing complex physicochemical and biological interactions across living systems.

Medicinal mushroom glycans, particularly β-glucans, represent some of the most biologically significant and extensively investigated active principles owing to their diverse structural organization and broad physiological activity (33). These polysaccharides are major constituents of fungal cell walls, which are primarily composed of glucans, glycoproteins, and smaller amounts of chitin arranged into complex supramolecular architectures (34). The dominant carbohydrate motifs are β-linked glucans, especially linear β-1,3-glucans containing smaller proportions of β-1,6- and β-1,4-linkages, which associate with chitin microfibrils and mannoprotein-rich networks to form highly heterogeneous and hydrated assemblies (34, 35). Importantly, the biological properties of mushroom glycans are strongly governed not only by their chemical composition, but also by branching patterns, molecular weight, conformational states, and assembly dynamics, all of which influence their interactions with immune receptors, gut microbiota, and metabolic signaling pathways. Through these interconnected mechanisms, mushroom-derived glycans have emerged as multifunctional bioactive molecules capable of modulating inflammation, oxidative stress, glucose homeostasis, and immune activity, positioning them at the center of current efforts to understand the molecular basis of the antidiabetic and cardiometabolic benefits associated with medicinal fungi (36). Despite their importance, however, the supramolecular organization of fungal glycans remains incompletely understood because these insoluble, amorphous, and structurally heterogeneous assemblies are difficult to characterize non-destructively at high resolution, and current structural models largely rely on indirect chemical and enzymatic approaches that often disrupt native polymer organization.

The hierarchical supramolecular organization of the *Ganoderma lucidum* (i.e., lingzhi or reishi) cell wall is illustrated in Figure 2. This model was proposed by Chen et al. based on nondestructive magic-angle spinning nuclear magnetic resonance (MAS NMR) spectroscopy (37), which enabled characterization of the native heterogeneous organization of fungal polysaccharide assemblies without disrupting the intact cell wall. The cell wall is a heterogeneous bilayer architecture composed of a highly hydrated and dynamic outer layer enriched in branched and structurally diverse glycans, and a comparatively rigid inner layer dominated by densely packed β-glucan and chitin-rich networks proximal to the plasma membrane (38). A hydration gradient across the cell wall is proposed based on water-edited NMR analyses, emphasizing increasing water mobility toward the exterior region. Other studies are also in line with the compositional and physicochemical heterogeneity of the fungal cell wall, with some degrees of variability in chemical composition present depending on the species (39).

**FIGURE 2:**
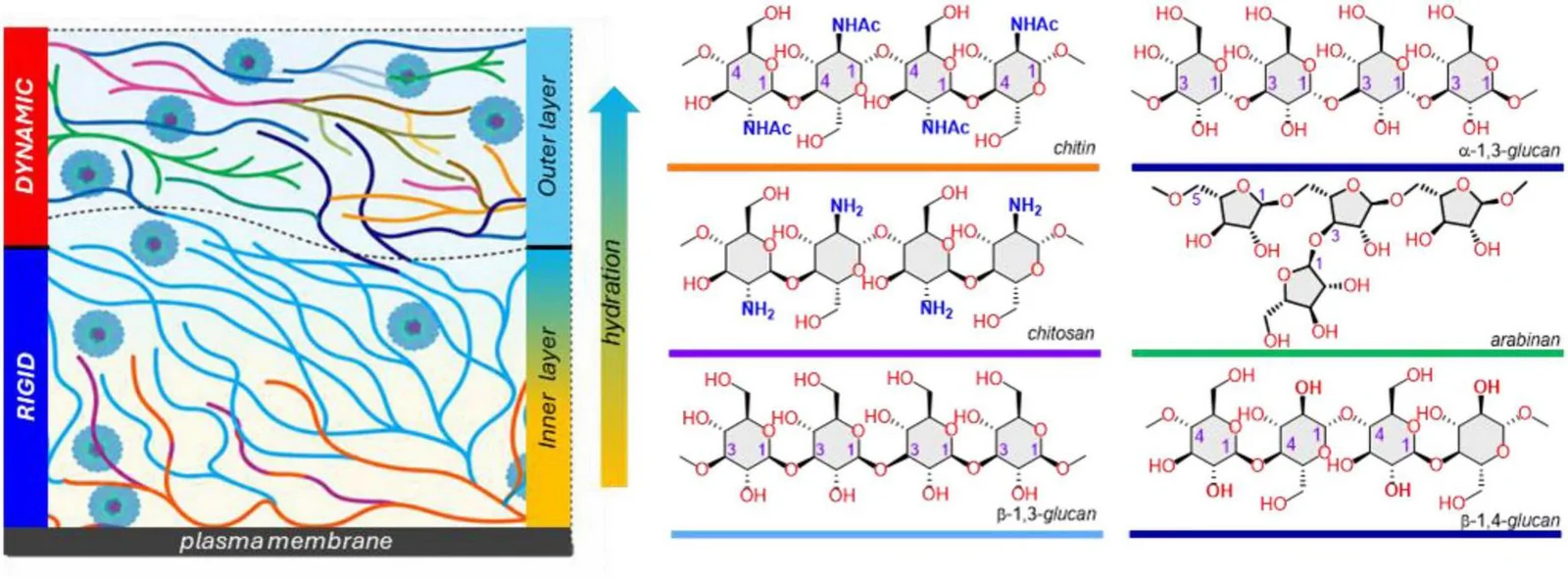
Schematic representation of the *Ganoderma lucidum* cell wall. Glycans are represented as colored lines, while proteins are shown as blue spheres. Representative polysaccharide structures are color-coded according to their proposed distribution within the cell wall.

Chitin and chitosans add important value to medicinal-mushroom bioactivity because they are not merely structural cell-wall polysaccharides, but functional, biocompatible biopolymer relevant as fungal fibers/bioactives that may support gut-barrier function, microbiota remodeling, anti-inflammatory signaling, lipid binding, and metabolic regulation. Cord-Landwehr and Moerschbacher recently introduced the concept of the “ChitoCode,” proposing that the biological functions of fungal chitins and chitosans are encoded not simply by composition, but by precise patterns of acetylation, molecular organization, and interactions with specific binding proteins (40). Hence, the fungal cell wall polysaccharides should be viewed as dynamic and information-rich biopolymers whose structural heterogeneity governs recognition, assembly, immune interactions, and biological activity. Chitin/chitosan function likely emerges from hierarchical supramolecular organization and biosynthetic patterning, drawing strong parallels to other biological coding systems. On the other hand, β-(1→3)/(1→6)-D-glucans influence macrophage activation, cytokine signaling, gut microbiota composition, and glucose metabolism, thereby contributing to improved insulin sensitivity and attenuation of chronic inflammation (33). Recent experimental evidence suggests that the biological activity of fungal glycans depends not only on their chemical composition, but also on their higher-order supramolecular organization and dynamic behavior, interestingly paralleling our very recent findings on the role of supramolecular motion in biomaterial function (41). It would be useful to further probe these mechanisms using *in vivo* models. Critically, these structural attributes are directly linked to antidiabetic function. The degree of solubility and molecular weight of β-glucans determine luminal viscosity and gel formation, which slow gastric emptying and intestinal glucose absorption and thereby blunt the postprandial glycemic response; the β-(1→3)/(1→6) branching pattern and the random-coil-to-triple-helix conformational state govern receptor recognition (e.g., Dectin-1- and complement receptor 3-mediated immunomodulation); and the fermentability of these polysaccharides by the gut microbiota yields short-chain fatty acids that stimulate GLP-1 secretion via free fatty acid receptors (FFAR2/FFAR3), improving insulin secretion, glucose tolerance, and gut-barrier integrity (42, 43). In this way, the supramolecular organization of the fungal cell wall provides a direct structural basis for its metabolic, immunological, and microbiota-mediated antidiabetic effects.

As evident in Figure 3a, lentinan from *Lentinula edodes*, known as shiitake mushrooms, exists as random coils (RC-LNT) and ordered triple-helical assemblies (TH-LNT) which are in dynamic equilibrium. In the context of amyloidogenic diseases, specifically T2DM, human islet amyloid polypeptide (hIAPP), normally co-secreted with insulin, undergoes polymerization into fibrillar aggregates driven by intermolecular β-sheet formation (44). Such aggregates are found toxic to islet β-cells and are hypothesized to contribute to β-cell dysfunction and loss during late-stage T2DM. As illustrated in Figure 3b, Xin et al. disclosed that TH-LNT can co-assemble with native hIAPP monomers and early assemblies, which inhibits the formation of pathological filamentous polymers associated with β-cell dysfunction and metabolic disease progression (45, 46). These findings reinforce that β-glucans act as adaptive supramolecular architectures rather than inert nutritional polysaccharides, with subtle structural variations profoundly influencing bioactivity, opening opportunities for T2DM therapies inspired by mushroom-derived polysaccharides (46).

**FIGURE 3:**
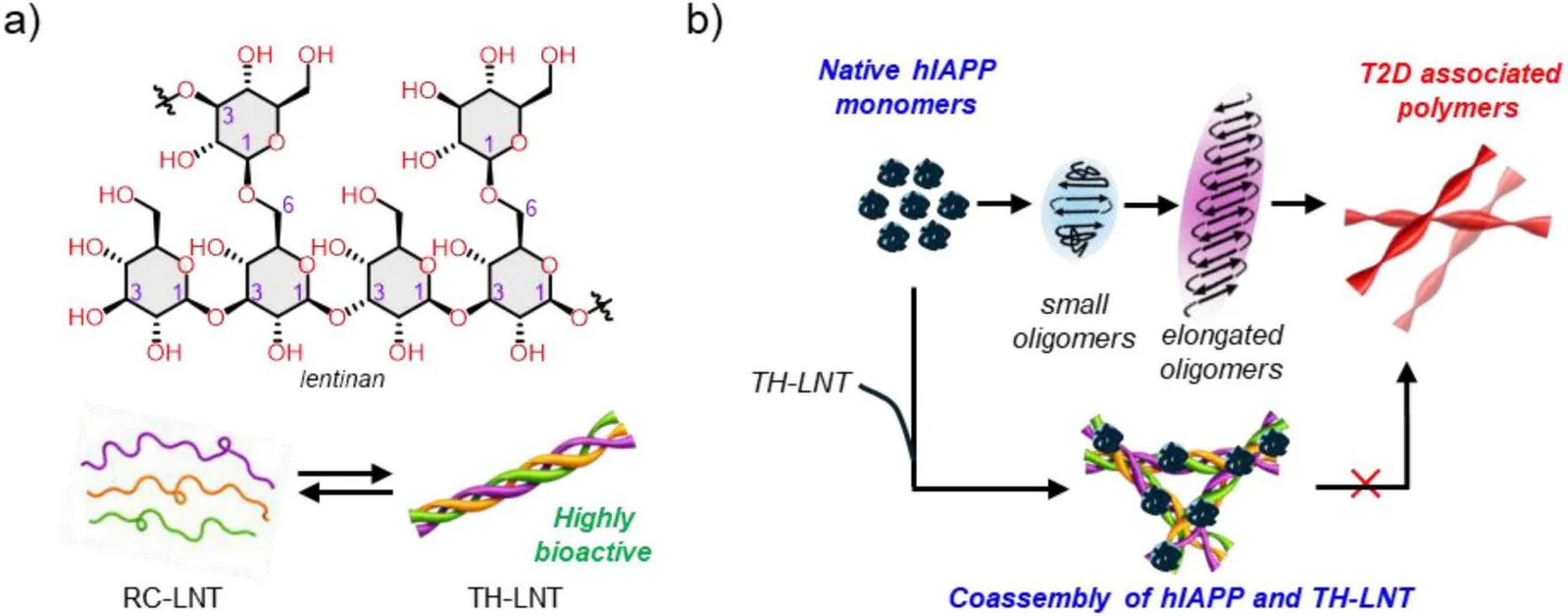
Proposed mechanism by which lentinan modulates pathological hIAPP aggregation through dynamic coassembly processes. **(a)** Chemical structure and dynamic equilibrium between random coil (RC) and ordered triple helical (TH) assemblies. **(b)** Dynamic interactions between TH-LNT and human islet amyloid polypeptide (hIAPP) redirecting peptide self-assembly pathways away from toxic fibrillar architectures associated with T2DM progression. This represents a proposed mechanism supported by preliminary experimental evidence and requiring further *in vivo* validation.

Medicinal mushrooms also contain structurally diverse low-molecular-weight metabolites with surprising bioactivities. Notably, lanostane-type triterpenoids from *Ganoderma lucidum*, comprising more than 300 chemically distinct structures identified to date, are emerging as important regulators of pathways associated with T2DM and metabolic dysfunction. Ganoderic acids A, C2, D, F, DM, X, and Y shown in Figure 4 have demonstrated activities ranging from α-glucosidase and aldose reductase inhibition to modulation of NF-κB, PI3K/Akt/mTOR, STAT3, and oxidative stress-related signaling (47). Particularly intriguing is ganoderic acid C2, which is under clinical investigation for diabetic complications and exhibits potent α-glucosidase inhibitory activity (48). Subtle variations in hydroxylation and carbonylation patterns and side-chain functionality generate structurally diverse ganoderic acids capable of modulating distinct biological pathways related to glucose homeostasis, inflammation, oxidative stress, and metabolic signaling. These compounds exemplify how small structural modifications within fungal triterpenoids can profoundly reshape their biological interaction landscapes and emergent bioactivity. Given the limited availability of these compounds, it comes as no surprise that more systematic evaluations of ganoderic acids in T2DM prevention and management are lacking.

**FIGURE 4:**
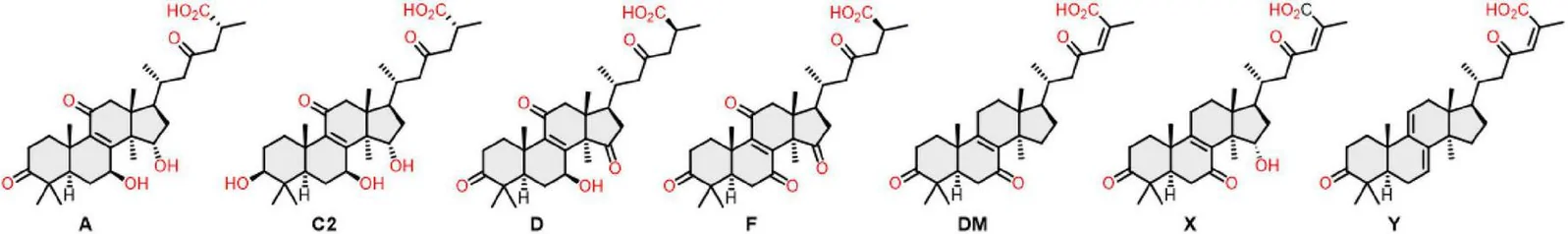
Structural formulae of highly bioactive Ganoderic acids.

Medicinal mushrooms contain a remarkably diverse repertoire of low-molecular-weight metabolites that extend far beyond triterpenoids and contribute significantly to their metabolic and redox-regulatory bioactivity (49). Diverse phenolic metabolites, including gallic, protocatechuic, caffeic, ferulic, and p-coumaric acids, together with fungal-specific polyphenols such as hispidin and inoscavin derivatives, contribute to antioxidant buffering, suppression of lipid peroxidation, modulation of inflammatory signaling, and protection against glucose-induced oxidative damage. Representative examples of these compounds, many of which have demonstrated antioxidant, anti-inflammatory, and glucose-regulatory activities, are depicted in Figure 5.

**FIGURE 5:**
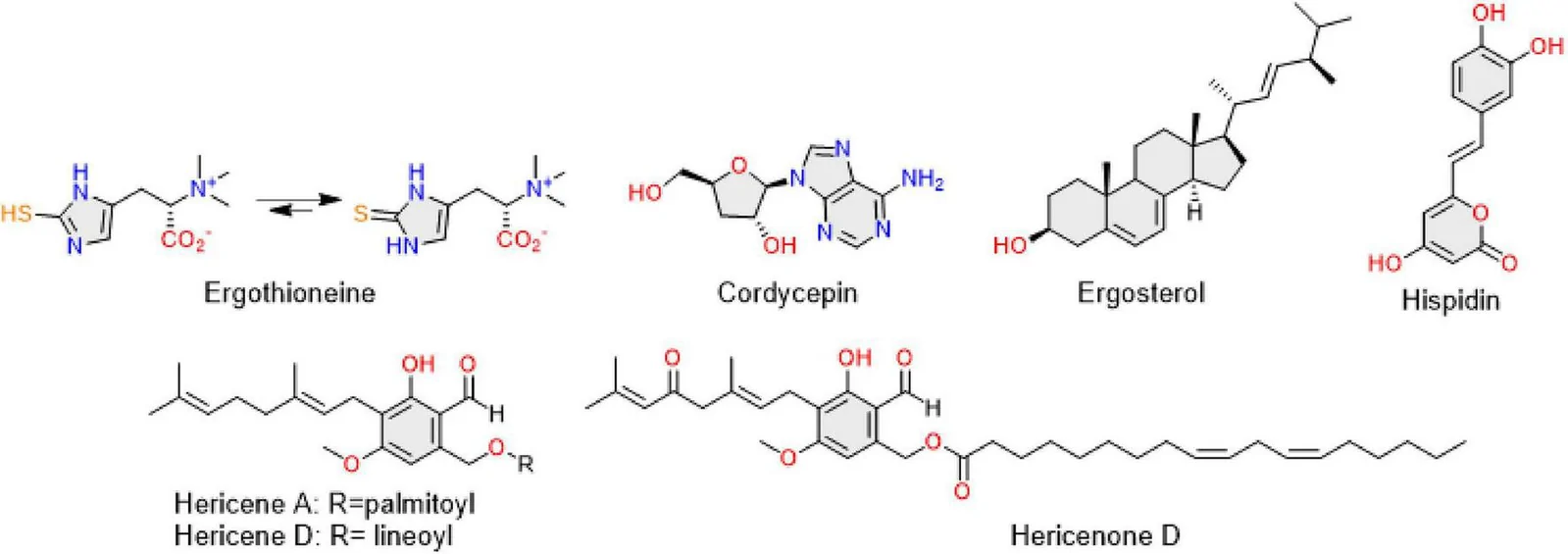
Secondary metabolites from medicinal mushrooms with demonstrated bioactivity.

Particular attention has recently been directed toward ergothioneine, an unusual sulfur-containing histidine derivative, abundant in mushrooms such as *Pleurotus ostreatus*, *Lentinula edodes*, and *Grifola frondosa* (50). Despite their distinct structures and biological origins, ergothioneine and ovothiol A illustrate how sulfur-containing histidine derivatives have emerged as redox-active metabolites in diverse organisms, potentially reflecting convergent chemical strategies for managing oxidative stress. Ergothioneine exists in thiol/thione equilibrium (Figure 5) and accumulates in tissues exposed to elevated oxidative stress through the highly selective OCTN1 transporter and has been proposed as a “longevity vitamin” because of its cytoprotective and anti-inflammatory properties. Mechanistically, ergothioneine may mitigate mitochondrial dysfunction, lipid peroxidation, endothelial injury, and inflammatory activation associated with obesity and T2DM progression (51).

Another important compound is cordycepin, a bioactive nucleoside analog (3′-deoxyadenosine) primarily isolated from *Cordyceps militaris* and related *Cordyceps* species, which has been shown to alleviate hyperglycemia-induced testicular damage by regulating downstream antioxidant enzyme activity through the SIRT1/Foxo3a signaling pathway (52). Molecular mechanistic analyses suggest that polysaccharides from *Hericium erinaceus* may help maintain glucose and lipid homeostasis in association with activation of the PI3K/Akt signaling pathway, thereby promoting glycogen synthesis and reducing lipid peroxidation in diabetic rats (53, 54). In addition, hericenone D, hericenes A and D isolated from the fruiting body, together with erinacerins Q-T derived from the mycelium, exhibit α-glucosidase inhibitory activity and may exert hypoglycemic effects by reducing carbohydrate absorption (53). Erinacerins Q-T also inhibit PTP-1B, a key negative regulator of insulin signaling, thereby improving glucose regulation and enhancing fat metabolism through modulation of insulin receptor phosphorylation and leptin signaling (55, 56).

Collectively, the chemistry of medicinal mushrooms reveals a highly multifunctional molecular landscape capable of simultaneously targeting multiple hallmarks of metabolic disease (20). Rather than acting through a single pharmacological mechanism, mushroom-derived bioactives appear to exert synergistic and pleiotropic effects across interconnected metabolic, inflammatory, redox, and immunological pathways, supporting their growing relevance as candidates for integrative nutritional strategies in T2DM prevention and metabolic health promotion (57).

## 4 Translational and clinical evidence

A literature search was conducted in PubMed to identify studies investigating the effects of mushrooms and fungal-derived bioactive compounds in T2DM (records up to March 2026). The search strategy included combinations of mushroom-related terms, including “mushrooms,” “fungi,” “medicinal mushrooms,” *Agaricus bisporus*, *Cordyceps militaris*, *Lentinula edodes*, *Phellinus linteus*, *Ganoderma lucidum*, *Cordyceps sinensis*, *Inonotus obliquus*, *Pholiota adiposa*, *Pleurotus ostreatus*, *Schizophyllum commune*, *Grifola frondosa*, *Trametes versicolor*, *Poria cocos*, and *Morchella esculenta*, combined with T2DM-related terms such as “type 2 diabetes,” “T2DM,” “hyperglycemia,” and “insulin resistance.” A total of 171 records were identified through database searching. After removal of duplicates and irrelevant records, full-text articles were assessed for eligibility according to predefined inclusion and exclusion criteria. Studies were included if they were original research articles investigating mushroom-derived extracts, polysaccharides, or isolated fungal bioactive compounds in experimental *in vivo* models or clinical studies involving T2DM. Studies were excluded if they were reviews, meta-analyses, epidemiological studies, *in silico* or computational studies, not focused on T2DM, not related to mushrooms or fungal bioactives, based on multi-component formulations where the specific contribution of mushrooms could not be clearly determined, letters to the editor, or unavailable as full text. Following screening, 58 studies were included in the qualitative synthesis.

A substantial body of evidence regarding medicinal mushrooms and their bioactive compounds in T2DM originates primarily from preclinical experimental models, whereas clinical investigations remain comparatively limited but increasingly informative. Together, the available literature provides a multi-layered perspective on the translational potential of fungal-derived bioactives in metabolic disease.

### 4.1 Preclinical evidence

Preclinical studies consistently report that mushroom-derived bioactive compounds exert antidiabetic effects across diverse experimental T2DM models. These include high-fat diet (HFD) combined with streptozotocin (STZ)-induced diabetic models, genetic models such as db/db, ob/ob, and KK-Ay mice, as well as chemically induced insulin resistance systems and alternative model organisms including zebrafish. Collectively, these experimental approaches recapitulate major features of human T2DM, including hyperglycemia, insulin resistance, dyslipidemia, and impaired glucose tolerance, thereby enabling evaluation of metabolic and molecular responses to mushroom-derived interventions (58–60).

As summarized in Table 1, a wide range of edible and medicinal mushroom species and their isolated bioactive fractions have been investigated. The most frequently studied taxa include *Ganoderma lucidum*, *Inonotus obliquus*, *Pleurotus ostreatus*, *Grifola frondosa*, *Lentinula edodes*, *Cordyceps* spp., *Trametes versicolor*, and *Hericium* spp. Across studies, bioactive interventions were administered predominantly as polysaccharide-rich extracts, although sterols, triterpenoids, glycopeptides, phenolic fractions, and ergosterol derivatives were also frequently evaluated, highlighting the chemical diversity of fungal-derived preparations.

**TABLE 1:** Preclinical animal studies investigating mushroom-derived bioactive compounds in experimental models of type 2 diabetes mellitus (grouped by experimental diabetes model).

Study	Mushroom/bioactives	Model	Treatment and duration	Key findings
Rehman et al. (58)	*Morchella esculenta* polysaccharides (MEP)	HFD + STZ-induced T2DM BALB/c mice	MEP 200–600 mg/kg/day, 4 weeks	↓ hyperglycemia; ↓ hyperlipidemia; ↑ insulin sensitivity ↑ gut microbiota modulation
Xiao et al. (80)	Termite fungus comb polysaccharides (TFCPs)	HFD + STZ-induced T2DM mice	300–500 mg/kg/day, 3–4 weeks	↓ glucose/lipids; ↑ insulin sensitivity; ↑ glycogen synthesis; ↑ gut microbiota modulation
Chen et al. (59)	GFP-N (heteropolysaccharide) from maitake mushrooms	T2DM mice	75–150 mg/kg, 28 days	↑ IRS-1/PI3K/GLUT4 signaling; ↓ JNK1/2
Wang et al. (60)	Polysaccharide IOPS from *Inonotus obliquus*	HFD + STZ-induced T2DM mice	300–900 mg/kg; 4 weeks	↓ glucose/lipids; ↑ antioxidant activity
Yang et al. (81)	*Armillariella tabescens* polysaccharides	HFD + STZ-induced T2DM mice	100–400 mg/kg, 4 weeks	↓ hyperglycemia; ↓ HOMA-IR; ↑ insulin sensitivity
Gong et al. (70)	Polysaccharide LNT-1 from *Lentinula edodes*	HFD + STZ-induced T2DM mice	50–200 mg/kg/day, 7 weeks	↓ oxidative stress
Sun et al. (82)	Mushroom polysaccharide NAP-3	T2DM mice model	100–200 mg/kg/day, 8 weeks	↑ glucose/lipid metabolism; ↑ GLP-1 signaling; ↑ gut microbiota remodeling
Lei et al. (83)	MT-α-glucan from *Grifola frondosa*	Murine T2DM model	150–450 mg/kg/day, 5 weeks	↓ glucose/lipids; ↑ insulin; ↑ antioxidant enzymes
Subramaniam et al. (71)	*Ganoderma neo-japonicum* polysaccharides (GNJP)	HFD-induced obese/T2DM C57BL/6J mice	50–200 mg/kg, 3 × weekly, 10 weeks	↓ weight gain; ↓ steatosis; ↓ hyperglycemia
Zhang et al. (84)	*Inonotus obliquus* extract	HFD + STZ-induced T2DM C57BL/6 mice	250–500 mg/kg/day oral gavage, 8 weeks	↓ glucose/lipids; ↑ glycogen ↓ TC ↓ TG
Wang et al. (85)	*Pleurotus eryngii* polysaccharide (PEP) *Boletus* polysaccharide (BP) *Lentinula edodes* polysaccharide (LEP) *Pleurotus ostreatus* polysaccharide (POP) *Agaricus bisporus* (ABP)	HFD + STZ-induced T2DM C57BL/6 mice	200 mg/kg of PEP/BP/LEP/POP, 4 weeks	Alleviated diabetic symptoms
Chou et al. (77)	Low-molecular-weight polysaccharide fraction (LIOP) from *Inonotus obliquus*	Type 2-like diabetic nephropathy mice (HFD + STZ)	300 mg/kg for 8 weeks	↓ renal fibrosis; ↓ inflammation; ↑ insulin sensitivity
Kan et al. (123)	*Cordyceps sinensis* ethanol extract from solid-state fermented mycelium	KK/HIJ type 2 diabetic mice fed a high-fat diet	300 mg/kg for 4 weeks	↑ HDL/LDL ratio; ↓ body weight gain trend; protected pancreatic β-cells from STZ-induced toxicity
Huang et al. (52)	*Cordyceps militaris* extracts/cordycepin	HFD + STZ-induced T2DM	10–20 mg/kg for 8 weeks	↓ testicular damage and apoptosis; ↑ antioxidant enzymes (Mn-SOD, CAT)
Liu et al. (124)	*Cordyceps militaris* extracts (CE); cordycepin (CCS)	HFD + STZ-induced T2DM	1–1.5 g/kg CE 25–50 mg/kg CCS 6 weeks	↓ glucose and lipid metabolism parameters ↓ oxidative stress biomarkers ↓ inflammatory cytokines
Lin et al. (78)	*Cordyceps militaris* (cultivated on *Ginkgo biloba* seeds)	ApoE knockout mice + Western diet (T2DM + diabetic nephropathy model)	111.3 μg, 8 weeks	↓ hyperglycemia, dyslipidemia
Yu et al. (79)	*Cordyceps militaris* (CmNo1 extract containing polysaccharides, cordycepic acid, adenosine, cordycepin)	HFD + STZ/nicotinamide-induced T2DM diabetic nephropathy mice	360 mg/kg/day, 8 weeks	↓ hyperglycemia; ↑ insulin sensitivity; ↓ serum creatinine and kidney hypertrophy
Wang et al. (93)	*Cordyceps militaris* polysaccharide fraction (AEPSa)	STZ-induced T2DM male Kunming ICR mice	100–400 mg/kg/day; 4 weeks	↓ FBG and serum lipids; ↑ IRS-1/IRS-2, PI3K, Akt, GLUT4 expression; ↓ insulin resistance and liver injury
Su et al. (98)	*Inonotus obliquus* polysaccharides	Kunming mice+HFD+STZ	150–600 mg/kg for 5 weeks	↓ LPS, TGs, TC; ↑ ZO-1, Ki67, MUC2; ↓ IL-6
Guo et al. (99)	Polysacharide from *Grifola frondosa*	HFD + STZ-induced T2DM in mice	900 mg/kg	↑ mRNA CYP7A1, BSEP
Zhao et al. (100)	Acidic-extractable polysaccharides (AE-PS) from *Cordyceps militaris*	HFD + STZ-induced T2DM mice	100 and 400 mg/kg AE-PS for 4 weeks	↓ blood glucose; ↓ serum lipids; ↓ lipid peroxidation; ↑ antioxidant enzyme activity
Chen et al. (104)	*Inonotus obliquus extract*	HFD + STZ-induced T2DM mice	150 mg/kg 5 weeks	↓ BW ↓ glucose ↓ LDL ↓ TG
Chen et al. (86)	*Ganoderma lucidum* polysaccharides (GLP)	HFD + STZ-induced T2DM rats	400 mg/kg/day orally, 4 weeks	↓ fasting glucose; ↓ insulin; ↑ gut microbiota modulation
Zhang et al. (67)	*Pleurotus ostreatus* polysaccharides (POP)	HFD + STZ-induced T2DM rats	100–400 mg/kg/day for 4 weeks	↓ hyperglycemia; ↑ GLUT4 translocation; ↑ antioxidant enzymes
Lo et al. (68)	*Trametes versicolor* extracellular polysaccharopeptides (TV-ePSP)	HFD + STZ-induced T2DM rats	0.1–1.0 g/kg/day, 4 weeks	↓ glucose; ↓ insulin resistance; ↑ antioxidant status
Razon et al. (87)	*Pleurotus ostreatus* + fenugreek	HFD + STZ-induced T2DM rats	5% diet supplementation for 8 weeks	↓ hyperglycemia; ↓ dyslipidemia; ↑ AMPK phosphorylation
Wang et al. (95)	Extracellular polysaccharopeptides (ePSP) from *Trametes versicolor*	HFD + STZ-induced T2DM rats	0.1–1.0 g/kg, 4 weeks	↓ postprandial glucose; ↓ hepatic lipids; ↑ liver metabolism
Feng et al. (97)	*Inonotus obliquus* extract	HFD + STZ-induced T2DM rats	50 and 100 mg/kg for 8 weeks	↓ cGMP ↓ BW ↓ glucose ↓ TC ↓ TG
Xian et al. (94)	Aqueous extract from *Coriolus versicolor* (CV)	HFD + STZ-induced T2DM rats	25–100 mg/kg/day, 4 weeks	↓ glucose; ↑ GLUT4 translocation
Wang et al. (90)	Ergosterol derivative (ETO)	HFD + STZ-induced T2DM rats	0.1–0.05 mmol/kg/day 4 weeks	↓ fasting blood glucose; ↓ dyslipidemia; ↑ antioxidant defense
Matsathit et al. (125)	Polysaccharide extract from *Schizophyllum commune*	HFD + STZ-induced T2DM rats	120–240 mg/kg body, 8 weeks	↓ glucose; ↑ insulin; ↑ GLUT2 expression
Xue et al. (91)	*Cordyceps militaris*/cordycepin	T2DM rats (STZ-induced)	10 mg/kg, 4 weeks	↑ vascular function; ↑ SIRT3; ↓ mitochondrial dysfunction
Dong et al. (126)	*Cordyceps militaris* water and alcohol extracts	HFD + STZ-induced T2DM rats	0.05–2 g/kg, for 3 weeks	↓ blood glucose; ↓ total cholesterol; ↓ triglycerides; ↑ antioxidant defense
Xiao et al. (88)	*Grifola frondosa* extract (GF5000)	STZ-induced diabetic Sprague–Dawley rats	112.5–675 mg/kg/day orally, 48 days	↑ insulin sensitivity; ↑ glucokinase; ↑ hepatic metabolism
Lo et al. (127)	Fruiting bodies (FB), submerged culture biomass (CM), and glucuronoxylomannan (GXM) from *Tremella mesenterica*	STZ + nicotinamide diabetic rats	FB, CM, or GXM 1 g/kg/day for 14 days	↓ glucose; ↑ OGTT response
Chen et al. (128)	Submerged-culture mycelia and broth of *Grifola frondosa*	STZ + nicotinamide diabetic rats	1 g/kg for 2 weeks	↓ glucose
Talpur et al. (96)	Niacin-bound chromium (NBC), fraction SX from *Grifola frondosa* (MSX), and hydroxycitric acid (HCA-SX from *Garcinia cambogia*)	Aged diabetic Zucker fatty rats	Oral gavage for 6 weeks	↓ blood pressure; ↓ oxidative stress; ↑ metabolic status
Xiong et al. (66)	Ergosterol (ERG) from *Pleurotus ostreatus*	KK-Ay mice genetically diabetic mice	ERG 60–120 mg/kg/day, oral gavage, 4 weeks	↓ fasting blood glucose; ↓ FBG; ↓ dyslipidemia; ↑ insulin sensitivity
Kobayashi et al. (72)	Ternatin from *Coriolus versicolor*	KK-Ay mice genetically diabetic mice	Continuous subcutaneous infusion via osmotic pump 8.5 or 17 nmol/day for 4 weeks	↓ hyperglycemia; ↓ hepatic lipogenesis
Hong et al. (69)	Maitake α-glucan from *Grifola frondosa* fruit body	KK-Ay mice genetically diabetic mice	150–450 mg/kg, twice a day, 2 weeks	↓ glucose/lipids; ↑ glycogen; ↑ antioxidant status
Yuan et al. (73)	FA polysaccharides from *Auricularia auricula-judae*	KK-Ay genetically diabetic mice	30 g/kg diet, 4 weeks	↓ glucose/insulin; ↑ glucose tolerance; ↑ glycogen
Yang et al. (74)	*Gomphidiaceae rutilus* polysaccharides	ob/ob mice	50 mg/kg/day for 4 weeks	↓ glucose; ↑ insulin sensitivity; ↓ hepatic lipid accumulation
Harada et al. (75)	Low-molecular-weight hot water extract (GGL) from *Grifola gargal*	ob/ob mice	2% dietary supplementation, 42 days	↓ glucose; ↓ triglycerides; ↓ adiposity
Yang et al. (76)	Polysaccharides from *Termitomyces albuminosus*	db/db mice	25–100 mg/kg/day for 8 weeks	↓ glucose/lipids; ↑ renal protection
Zheng et al. (129)	*Cordyceps cicadae* polysaccharides (CCPs)	db/db mice	75–300 mg/kg/day for 12 weeks	↓ glomerular mesangial expansion and sclerosis; ↓ apoptosis; ↓ inflammation; ↓ oxidative stress
Lin et al. (92)	*Betulinic acid (BA) from I. obliquus*	db/db mice	45 days	↓ BW; ↓ glucose; ↓ lipid levels
Wang et al. (54)	Polysaccharide HCP-2b from *Hericium coralloides*	T2DM zebrafish model	10–500 μg/ml, 48 h	↓ hyperglycemia; ↓ oxidative stress
Liu et al. (124)	Water-soluble lyophilized micelle from *Cordyceps militaris*	HFD + STZ-induced T2DM mice	1–1.5 g/kg for 6 weeks	↓ IL-6; ↓ TNF-α ↓oxidative stress
Heriansyah et al. (130)	Polysaccharide peptide from *Ganoderma lucidum*	HFD + STZ-induced T2DM rats	50–300 mg/kg for 4 weeks	↓ H_2_O_2_
Liu et al. (131)	Commercial *Cordyceps sinensis*	HFD + STZ-induced T2DM rats	0.4 and 1 mg/kg for 4 weeks	↓ glycemia; ↓ fasting insulin
Ye et al. (132)	*Inonotus obliquus* methanol extract	Murine T2DM model	600 mg/kg for 8 weeks	↓ hyperglycemia; ↓ TNF-α, IL-1β, IL-6; ↑ IL-10
She et al. (89)	Polysacharide from *Grifola frondosa*	HFD + STZ-induced T2DM rats	1.5 mg/kg 4 weeks	↓ HOMA-IR ↓ fasting glucose ↓ LPS, D-LA, DAO ↑ ZO-1, occludin ↓ TNF-α,NF-kB

↑, increase/upregulation; ↓, decrease/downregulation.

Despite variability in experimental design, dosing regimens, and treatment duration, preclinical findings show a high degree of consistency in metabolic outcomes. Across HFD/STZ, genetic, and chemically induced models, mushroom-derived interventions were repeatedly associated with improvements in fasting blood glucose, oral glucose tolerance, insulin sensitivity indices (including the homeostatic model assessment of insulin resistance, HOMA-IR), and lipid parameters such as total cholesterol, triglycerides, and LDL-cholesterol. In several studies, additional beneficial effects were observed on body weight regulation, hepatic lipid accumulation, and diabetic complications including nephropathy and tissue-specific injury (60–75).

Among the experimental models, HFD/STZ systems were the most frequently employed, reflecting their ability to mimic combined insulin resistance and partial β-cell dysfunction. Genetic models, particularly db/db, ob/ob, and KK-Ay mice, further confirmed the metabolic effects of mushroom-derived compounds in obesity-driven and hereditary forms of metabolic dysfunction (65, 69, 72–76). In addition, specialized models such as diabetic nephropathy models and zebrafish systems extended the evidence base beyond classical metabolic endpoints, suggesting broader systemic relevance (54, 77–79).

Interventions were generally administered orally over short-to-intermediate durations (approximately 2–12 weeks), with doses ranging widely depending on extract type and species. Notably, polysaccharide-rich preparations from *Ganoderma*, *Inonotus*, *Pleurotus*, and *Grifola* species represented the most extensively studied category and were consistently associated with improved systemic glucose and lipid homeostasis across multiple experimental platforms (59, 67–71, 80–89). In parallel, non-polysaccharide compounds, including ergosterol derivatives, betulinic acid, and cordycepin-containing extracts, also demonstrated significant metabolic benefits, underscoring the contribution of both primary and secondary fungal metabolites (52, 65, 90–92).

Overall, the preclinical evidence summarized in Table 1 supports the concept that medicinal mushrooms exert consistent antidiabetic effects across diverse experimental systems. However, these findings should be interpreted with caution, as most preclinical studies employ heterogeneous models, relatively high doses, and short treatment durations, and are subject to variability in preparation and limited methodological standardization and risk-of-bias assessment (see Section “6 Translational relevance, limitations, and caveats”). The consistency of metabolic improvements across species, models, and bioactive classes strengthens the translational rationale for further investigation, while also highlighting the importance of standardization and mechanistic clarification in future studies.

### 4.2 Clinical evidence

In contrast to the extensive preclinical literature, clinical studies investigating mushroom-derived bioactive compounds in patients with T2DM remain limited in number but provide important translational insights. As summarized in Table 2, available clinical evidence includes randomized controlled trials (RCTs), as well as intervention studies conducted in both diabetic and mixed metabolic populations, primarily evaluating *Agaricus*, *Pleurotus*, and *Cordyceps* species and their bioactive preparations.

**TABLE 2:** Clinical studies investigating mushroom-derived bioactive compounds in patients with type 2 diabetes mellitus.

Study	Mushroom (bioactives)	Design and population	Intervention and duration	Key findings
Hashemi Yusefabad et al. (61)	*Agaricus bisporus* hot air-dried powder (HAD-WBM)	RCT, double-blind, placebo-controlled; T2DM adults 20–50 y; *n* = 44	16 g/day HAD-WBM vs. cornstarch placebo, 8 weeks	↓ fructosamine; ↓ FBS; ↓ LDL cholesterol; ↓ HOMA-IR, (significant within-group, *p* < 0.05)
Hsu et al. (62)	*Agaricus blazei* Murill extract (ABM extract, polysaccharide-rich)	RCT, double-blind, placebo-controlled; T2DM on gliclazide + metformin; *n* = 72	1500 mg/day ABM extract vs. cellulose placebo (adjunct), 12 weeks	↓ HOMA-IR; ↑ adiponectin; ↑ insulin sensitivity, (significant vs. placebo, *p* < 0.05)
Jayasuriya et al. (63)	*Pleurotus ostreatus* + *Pleurotus cystidiosus* (freeze-dried sporophore powder, SFDP preparation)	Acute single-dose study; healthy volunteers + diet-controlled T2DM; *n* = 14 (T2DM)	50 mg/kg body weight, single oral dose with glucose load	↓ fasting and postprandial glucose; ↑ insulin; ↑ glucose utilization; ↑ GK; ↓ GSK; ↑ glycogen synthesis, (significant, *p* < 0.05)
Kai et al. (64)	*Cordiceps* (Dongchongxiacao/corbrin capsule)	RCT, 3 arms (basic *n* = 41; standard *n* = 39; intensive *n* = 40); T2DM with renal insufficiency undergoing coronary angiography; *n* = 120	2 g (standard) or 3 g (intensive) capsules × 3/day, 3 days before and after angiography	↓ incidence of contrast-induced nephropathy (11/120; 9.17%; greater reduction with intensive dose); ↓ urinary kidney-injury and inflammatory biomarkers, (significant vs. basic treatment)
Klupp et al. (65)	*Ganoderma lucidum* (Reishi/Lingzhi; polysaccharides, triterpenoids) ± *Cordyceps sinensis* (polysaccharides and nucleosides)	RCT, double-blind, placebo-controlled, parallel 3-arm; adults with T2DM and metabolic syndrome; *n* = 84	3 g/day *G. lucidum* ± *C. sinensis* (8 capsules/day) vs. placebo, 16 weeks	No significant effect on HbA1c, fasting plasma glucose, blood pressure, triglycerides, total cholesterol, HDL/LDL, or BMI, (all non-significant, *p* > 0.05)

↑, increase/upregulation; ↓, decrease/downregulation.

Overall, clinical interventions have focused predominantly on polysaccharide-rich extracts or whole mushroom preparations administered orally over short-to-intermediate durations ranging from several days to 12 weeks. Across studies, evaluated outcomes primarily included glycemic control parameters, insulin sensitivity indices, lipid profile markers, and selected renal and inflammatory biomarkers.

Hashemi et al. reported that supplementation with hot air-dried *Agaricus bisporus* powder was associated with significant reductions in fructosamine, fasting blood sugar (FBS), LDL cholesterol, and HOMA-IR compared to baseline values, suggesting improved short-term glycemic control and lipid metabolism in patients with T2DM (61). Similarly, Hsu et al. reported that administration of *Agaricus blazei* Murill polysaccharide-rich extract, as an adjunct to standard antidiabetic therapy, improved insulin sensitivity and significantly increased adiponectin levels while reducing HOMA-IR, indicating enhanced systemic insulin responsiveness (62).

In addition, Jayasuriya et al. observed that *Pleurotus ostreatus* and *Pleurotus cystidiosus* preparations improved fasting glucose levels and insulin response in both healthy individuals and patients with T2DM. These effects were accompanied by enhanced glucose utilization and glycogen synthesis, suggesting modulation of peripheral glucose metabolism and insulin-related enzymatic activity (63).

Beyond glycemic regulation, emerging evidence also suggests potential renoprotective effects. In a clinical study involving patients with T2DM undergoing coronary angiography, *Cordyceps* supplementation was associated with a reduced incidence of contrast-induced nephropathy, as well as decreased urinary biomarkers of kidney injury and inflammation, indicating potential protective effects on renal function in high-risk clinical settings (64).

However, not all clinical investigations have confirmed the beneficial effects observed in experimental models. In a prospective, randomized, double-blind, placebo-controlled clinical trial, Klupp et al. evaluated the effects of *Ganoderma lucidum* supplementation, alone or combined with *Cordyceps sinensis*, in patients with T2DM and metabolic syndrome. Despite promising preclinical evidence suggesting antihyperglycemic, hypolipidemic, and cardioprotective properties of these mushrooms, 16 weeks of supplementation with *G. lucidum*-based preparations did not result in significant improvements in glycated hemoglobin (HbA1c), fasting plasma glucose, blood pressure, lipid profile, inflammatory markers, or other components of metabolic syndrome compared with placebo. The intervention was generally well tolerated, with predominantly mild adverse events. Importantly, this study highlights the challenges associated with translating mechanistic and preclinical findings into clinically meaningful outcomes and emphasizes the influence of factors such as bioactive compound standardization, dosage, treatment duration, and study power on clinical efficacy (65).

Collectively, current clinical evidence suggests that mushroom-derived bioactive compounds may have beneficial effects on metabolic regulation in T2DM, particularly through improvements in glycemic control, insulin sensitivity, lipid metabolism, and protection against diabetes-associated complications. However, these effects remain inconsistent across clinical trials, with some well-designed studies failing to demonstrate significant metabolic benefits. The interpretation of available evidence is limited by small sample sizes, short intervention periods, variability in mushroom species and extraction methods, differences in bioactive compound content, and the absence of standardized preparations. Therefore, larger, long-term, and rigorously controlled clinical trials are required to establish the therapeutic potential and clinical relevance of medicinal mushrooms in T2DM management.

Notably, the available human data derive almost exclusively from participants with already established T2DM; consequently, current evidence speaks to adjunctive management rather than primary prevention.

### 4.3 Integrative interpretation of translational evidence

Taken together, the available evidence reveals broadly consistent but still incomplete translational trajectory linking preclinical findings with emerging and mixed clinical observations on the metabolic effects of medicinal mushrooms in T2DM. While preclinical studies report consistent improvements in glucose homeostasis, lipid metabolism, and insulin sensitivity across multiple experimental models, clinical studies provide preliminary and inconsistent evidence of beneficial effects on glycemic control, insulin resistance, and selected cardiometabolic and renal outcomes.

Despite differences in study design, population characteristics, intervention duration, and mushroom preparations, a shared pattern emerges across both experimental and clinical settings. Specifically, mushroom-derived interventions are associated, in most though not all studies, with improvements in systemic metabolic regulation, including reductions in fasting and postprandial glucose levels, improved insulin sensitivity indices, and favorable modulation of lipid profiles. This convergence supports the translational relevance of preclinical observations and suggests that key metabolic effects may be at least partially preserved in humans. Nevertheless, this pattern is not uniform, as some well-controlled trials (65) reported no significant metabolic benefit, underscoring that the translational signal remains provisional.

However, the strength of this translational alignment is moderated by several important limitations. Preclinical studies predominantly rely on short-term interventions in highly controlled experimental models that only partially recapitulate the complexity and heterogeneity of human T2DM. In parallel, clinical studies remain limited in number, sample size, and duration, with substantial variability in mushroom species, bioactive composition, and dosing strategies. Moreover, mechanistic endpoints such as oxidative stress markers, inflammatory mediators, and gut microbiota composition are rarely assessed in clinical settings, restricting direct mechanistic mapping between experimental and human data.

Nevertheless, the overall directionality of evidence supports a coherent biological concept: medicinal mushrooms exert pleiotropic metabolic effects through multi-target modulation of interconnected pathways governing glucose metabolism, insulin signaling, lipid handling, and systemic metabolic resilience. This multi-axis activity aligns with the complex and network-based pathophysiology of T2DM, in which metabolic dysfunction arises from integrated failure across multiple regulatory systems rather than a single molecular defect.

From a translational perspective, these findings collectively support the continued development of medicinal mushrooms as functional food-based adjunct strategies for metabolic disease prevention and management. However, the current evidence base remains insufficient to support clinical standardization or therapeutic positioning, emphasizing the need for large-scale, well-controlled, and mechanistically informed clinical trials integrating metabolic, inflammatory, and microbiome-related endpoints.

## 5 Systems-level mechanistic integration of medicinal mushroom bioactivity in T2DM

The antidiabetic activity of medicinal mushrooms is increasingly understood as a systems-level phenomenon rather than a set of isolated pharmacological actions. Across experimental systems, mushroom-derived bioactive compounds converge on a limited number of core regulatory axes, insulin-energy sensing signaling, redox regulatory systems, immune-metabolic crosstalk, and gut-derived endocrine signaling, that are tightly interconnected in the pathophysiology of T2DM. Rather than acting as single-target agents, these compounds appear to function as network-active metabolic modulators capable of partially counteracting the dysregulated physiology characteristic of T2DM.

Because many studies infer pathway involvement from phosphorylation, gene-expression, or biomarker changes, the mechanistic pathways discussed below should be interpreted as candidate or associated mechanisms unless functional validation is explicitly reported.

### 5.1 Insulin-AMPK signaling network as the core metabolic hub

A central and consistently observed mechanistic feature across studies is the modulation of the insulin signaling cascade and its integration with cellular energy-sensing pathways, particularly AMPK. Collectively, the available evidence indicates that mushroom-derived bioactive compounds act as multi-target regulators converging on the insulin-AMPK signaling network, which represents a key metabolic hub controlling glucose uptake, lipid metabolism, and overall insulin sensitivity in T2DM.

Across multiple experimental systems, polysaccharides and bioactive fractions from *Pleurotus*, *Grifola*, *Inonotus*, *Ganoderma*, *Cordyceps*, and *Trametes* species consistently enhanced insulin signaling through activation of the IRS-1/PI3K/Akt axis and downstream GLUT4 translocation, resulting in increased peripheral glucose uptake and improved glycemic control (59, 63, 67, 80, 84, 90, 93, 94). For example, polysaccharides from *Grifola frondosa* and *Pleurotus ostreatus* were shown to stimulate IRS-1/PI3K/GLUT4 signaling and improve glycogen synthesis while simultaneously suppressing inhibitory signaling pathways such as JNK1/2 and GSK-3, thereby improving insulin responsiveness at the cellular level (59, 63). Similarly, *Inonotus obliquus* polysaccharides and *Trametes versicolor* extracts enhanced PI3K/Akt-mediated glucose transport and improved insulin sensitivity in insulin-resistant models (84, 94).

In parallel, AMPK emerges as a critical energy-sensing node that integrates metabolic stress signals with systemic glucose and lipid homeostasis. Increased AMPK phosphorylation has been repeatedly reported following treatment with mushroom-derived polysaccharides and polyphenol-rich extracts, leading to increased glucose utilization, enhanced fatty acid oxidation, and suppression of hepatic gluconeogenesis (74, 87, 95, 96). In particular, *Agaricus blazei Murill* extract was associated with increased adiponectin levels and improved insulin sensitivity (62), effects that may intersect with AMPK-regulated metabolic signaling. Likewise, co-treatment with *Pleurotus ostreatus* and *Trigonella foenum-graecum* further enhanced AMPK phosphorylation and has been associated with improved lipid metabolic profiles, suggesting synergistic regulation of energy homeostasis (87).

Importantly, these insulin- and AMPK-mediated effects are likely closely interconnected rather than independent processes. Activation of PI3K/Akt signaling promotes GLUT4 translocation and glucose uptake, while AMPK activation enhances insulin sensitivity and suppresses lipogenesis through downstream targets such as ACC and SREBP-1c (72, 86). This coordinated regulation contributes to reduced hepatic glucose output, improved peripheral glucose disposal, and attenuation of dyslipidemia, collectively addressing both glycemic and lipid abnormalities characteristic of T2DM.

Additionally, several mushroom-derived triterpenes and sterol-related compounds contribute to metabolic regulation through complementary mechanisms, including inhibition of carbohydrate-digesting enzymes (α-glucosidase and α-amylase), thereby reducing postprandial glucose excursions and indirectly supporting insulin signaling homeostasis (47, 48). Cordyceps cicadae polysaccharides further extend this regulatory network by enhancing insulin signaling and improving glucose uptake in peripheral tissues by affecting IRS-1/PI3K/Akt/GLUT4 pathways (93).

Overall, the evidence summarized in Table 3 supports the concept that the insulin-AMPK signaling network represents a central mechanistic hub through which medicinal mushrooms exert pleiotropic antidiabetic effects. Rather than acting through isolated pathways, these bioactive compounds coordinate multiple nodes of insulin signaling, energy sensing, and metabolic regulation, thereby supporting systemic glucose and lipid homeostasis in experimental models of T2DM.

**TABLE 3:** Key metabolic mechanisms of mushroom-derived bioactive compounds in T2DM.

Compound	Source	Metabolic role	Reference
β-glucans Polyphenols	*Agaricus bisporus*	↓ glycemia; ↓ HOMA-IR; ↑ insulin sensitivity signaling ↓ LDL-C; improved lipid metabolism AMPK/ACC	Hashemi Yusefabad et al. (61)
Ergosterol	*Pleurotus ostreatus*	↑ glucose uptake; ↑ insulin sensitivity; ↓ dyslipidemia	Xiong et al. (66)
*Pleurotus ostreatus* polysaccharides (POP)	*Pleurotus ostreatus*	↑ GLUT4 translocation; ↑ glycogen storage PI3K/Akt; PKC; GLUT4 translocation	Zhang et al. (67)
*Inonotus obliquus* polysaccharides (IOPS)	*Inonotus obliquus*	↓ hyperglycemia; ↓ insulin resistance PI3K/Akt/GLUT4 signaling pathway	Zhang et al. (84)
GFP-N polysaccharide	*Grifola frondosa*	↑ glucose uptake and insulin signaling IRS-1/PI3K/GLUT4 activation; JNK1/2 inhibition	Chen et al. (59)
TFCPs	Termite fungus comb polysaccharides	↑ insulin sensitivity; ↑ glycogenesis; ↓ gluconeogenesis	Xiao et al. (80)
Ergosta-4,6,8(14),22-tetraen-3-one (ETO)	*Pholiota adiposa*	↓ glucose/lipids; improved OGTT IRS1/PI3K/AKT signaling	Wang et al. (90)
CV aqueous extract	*Coriolus versicolor*	↑ glucose uptake; ↓ insulin resistance PI3K/Akt; p38 MAPK; GLUT4	Xian et al. (94)
ePSP (extracellular polysaccharopeptides)	*Trametes versicolor*	↓ gluconeogenesis; ↑ glucose transport IR/IRS/PI3K/Akt; AMPK	Wang et al. (95)
*Pleurotus ostreatus* and *Pleurotus cystidiosus*	*Pleurotus ostreatus*, *P. cystidiosus*	↓ fasting/postprandial glucose; ↑ glycogen synthesis; ↑ GK; ↓ GSK-3	Jayasuriya et al. (63)
ABM extract	*Agaricus blazei* Murill	↓ HOMA-IR; ↑ adiponectin; ↑ insulin sensitivity; adiponectin/AMPK signaling	Hsu et al. (62)
Bioactive phytochemicals	*Pleurotus ostreatus* + *Trigonella foenum-graecum*	↓ hyperglycemia; improved lipid metabolism; AMPK activation	Razon et al. (87)
*Ganoderma neo-japonicum* polysaccharides (GNJP)	*Ganoderma neo-japonicum*	↓ hyperglycemia; improved lipid metabolism	Subramaniam et al. (71)
AGRP total polysaccharides	*Gomphidiaceae rutilus*	↑ glucose uptake, ↓ blood glucose, ↑ insulin sensitivity; AMPK activation	Yang et al. (74)
(-)-Ternatin	*Coriolus versicolor*	↓ hyperglycemia; ↓ hepatic lipogenesis ↓ SREBP-1c signaling	Kobayashi et al. (72)
Water-soluble fraction (SX)	Maitake mushroom (SX)	↓ insulin resistance; ↓ fasting glucose	Talpur et al. (96)
Triterpenes	*Polysaccharides Ganoderma lucidum*	↓α-glucosidase/α-amylase activity; ↓ postprandial glucose; carbohydrate digestion inhibition; PI3K/Akt	Chen et al. (86)
Fruiting bodies (FB), submerged culture biomass (CM), or the acidic polysaccharide glucuronoxylomannan (GXM)	*Tremella mesenterica*	↓ postprandial glucose; improved OGTT; glycemic regulation pathways	Lo et al. (127)
*Cordyceps cicadae* spore powder polysaccharides	*Cordyceps cicadae*	↑ insulin signaling (IRS-1 ↑ PI3K/Akt ↑ GLUT4); ↑ glucose uptake; ↓ insulin resistance; ↓ hyperglycemia; ↓ dyslipidemia	Wang et al. (93)

↑, increase/upregulation; ↓, decrease/downregulation.

### 5.2 Redox homeostasis as a central regulatory axis

Oxidative stress represents a central pathogenic amplifier in T2DM, linking hyperglycemia, insulin resistance, mitochondrial dysfunction, and inflammatory activation into a self-perpetuating metabolic loop (Table 4). Across preclinical studies, mushroom-derived bioactive compounds consistently show a capacity to improve redox balance by enhancing endogenous antioxidant defense systems and suppressing oxidative damage markers. This coordinated regulation is characterized by increased activity of key enzymatic antioxidants, including superoxide dismutase (SOD), catalase (CAT), glutathione peroxidase (GSH-Px), and elevated reduced glutathione (GSH), alongside a marked reduction in lipid peroxidation products such as malondialdehyde (MDA) (68–70, 83, 86, 97).

**TABLE 4:** Secondary mechanisms of oxidative stress modulation of mushroom-derived bioactive compounds in T2DM.

Compound	Source	Redox role	Reference
*Ganoderma lucidum* polysaccharides (GLP)	*Ganoderma lucidum*	↓ oxidative stress (↓ MDA, ↑ SOD, CAT, GSH-Px), ↓ inflammatory markers	Chen et al. (86)
Extracellular polysaccharopeptides (TV-ePSP)	*Trametes versicolor*	↑ SOD/glutathione; ↓ lipid peroxidation	Lo et al. (68)
MT-α-glucan	*Grifola frondosa*	↑ SOD/GSH-Px/GSH; ↓ MDA	Hong et al. (69)
MT-α-glucan	*Grifola frondosa*	↓ oxidative/nitrosative stress (↓ MDA, NO, iNOS); ↑ antioxidant defenses (↑ SOD, GPx, GSH)	Lei et al. (83)
LNT-1 polysaccharide	*Lentinula edodes*	↓ oxidative stress; Nrf2/HO-1 signaling	Gong et al. (70)
AT polysaccharides	*Armillariella tabescens*	↓ MDA; ↑ antioxidant capacity ↓ TXNIP/NLRP3; ↑ AMPK/ACC	Yang et al. (81)
Split gill mushroom polysaccharides	*Schizophyllum commune*	↓ pancreatic oxidative stress	Matsathit et al. (125)
Inonotus obliquus extract	*Inonotus obliquus*	↑ SOD/CAT/GPx; ↓ MDA	Zhang et al. (84)
Ergothioneine	*Agaricus bisporus*	Antioxidant thiol protection	McElwain et al. (50)
Cordycepin	*Cordyceps militaris*	SIRT3-mediated mitochondrial signaling	Xue et al. (91)
Cordycepin	*Cordyceps militaris*	Activation of SIRT1/Foxo3a pathway; ↑ Mn-SOD and CAT antioxidant enzymes; ↓ Oxidative stress-induced testicular injury	Huang et al. (52)

↑, increase/upregulation; ↓, decrease/downregulation.

Mechanistically, activation of the Nrf2/HO-1 signaling axis emerges as a central upstream regulatory pathway through which multiple mushroom-derived polysaccharides exert their antioxidant effects. In particular, *Lentinula edodes* polysaccharide (LNT-1) has been shown to attenuate oxidative stress via Nrf2/HO-1 activation, reinforcing its role as a master regulator of cellular redox adaptation (70). Similarly, polysaccharides from *Armillariella tabescens* not only reduce MDA levels but also modulate redox-sensitive inflammatory and metabolic nodes, including TXNIP/NLRP3 signaling, while concurrently enhancing AMPK/ACC activity, thereby linking oxidative stress control to energy metabolism regulation (81).

In several experimental models, improvements in antioxidant capacity are directly associated with preservation of pancreatic and peripheral metabolic function. For example, *Grifola frondosa*-derived MT-α-glucans significantly enhance SOD, GSH-Px, and GSH levels while reducing MDA and nitrosative stress markers, correlating with improved glycemic control and insulin sensitivity (69, 83). These findings support the concept that redox regulation is not merely a downstream consequence of improved glucose metabolism, but rather an upstream determinant of insulin signaling efficiency and β-cell integrity.

Beyond polysaccharides, low-molecular-weight fungal metabolites contribute substantially to redox regulation. Ergothioneine, a sulfur-containing antioxidant abundant in mushrooms, exerts thiol-based cytoprotective effects and provides sustained protection against oxidative injury, particularly in metabolically sensitive tissues (50). In parallel, cordycepin from *Cordyceps militaris* activates SIRT3- and SIRT1/Foxo3a-dependent pathways, enhancing mitochondrial antioxidant defenses, including Mn-SOD and CAT, thereby improving mitochondrial redox homeostasis and reducing oxidative stress–induced tissue damage (52, 91). These mitochondrial effects are particularly relevant given the central role of mitochondrial dysfunction in insulin resistance and T2DM progression.

Importantly, redox modulation is tightly integrated with inflammatory and metabolic signaling networks. In several models, improved antioxidant status coincides with suppression of pro-oxidant and pro-inflammatory mediators, suggesting disruption of the oxidative stress–inflammation feed-forward loop that exacerbates insulin resistance (81, 86). This interconnected regulation highlights redox homeostasis as a central mechanistic hub through which medicinal mushrooms exert systemic metabolic benefits.

Collectively, the evidence indicates that mushroom-derived bioactive compounds regulate redox homeostasis through a multi-level mechanism involving enzymatic antioxidant enhancement, suppression of lipid peroxidation, activation of Nrf2-centered stress response pathways, and reinforcement of mitochondrial defense systems. This integrated redox control not only preserves cellular integrity but also stabilizes insulin signaling and metabolic function, positioning oxidative stress modulation as a core therapeutic axis in the context of T2DM prevention and management.

### 5.3 Immunometabolic reprogramming and inflammatory attenuation

Chronic low-grade inflammation is a fundamental driver of insulin resistance and metabolic deterioration in T2DM, functioning as a key link between nutrient overload, oxidative stress, and impaired insulin signaling. Across preclinical studies, mushroom-derived bioactive compounds consistently show immunomodulatory effects characterized by suppression of pro-inflammatory cytokines and attenuation of canonical inflammatory signaling pathways (Table 5). These effects include reduced levels of IL-1β, TNF-α, and IL-6, alongside inhibition of NF-κB activation and related downstream inflammatory cascades (58, 75, 77, 79, 95).

**TABLE 5:** Secondary mechanisms of anti-inflammatory effects modulation of mushroom-derived bioactive compounds in T2DM.

Compound	Source	Anti-inflammatory role	Reference
*Morchella esculenta* polysaccharide (MEP)	*Morchella esculenta* (morel mushroom)	↓ IL-1β/TNF-α/IL-6; improved intestinal barrier integrity	Rehman et al. (58)
Low-molecular-weight *I. obliquus* polysaccharides (LIOP)	*Inonotus obliquus*	↓ oxidative/inflammatory stress via reduced NF-κB activation; ↓ NF-κB/TGF-β1 signaling pathway (anti-fibrotic, anti-inflammatory)	Chou et al. (77)
GGL	*Grifola gargal*	↓ IL-6	Harada et al. (75)
PTA polysaccharides	*Termitomyces albuminosus*	↓ renal oxidative stress/inflammation; ↓ NF-κB signaling	Yang et al. (76)
CmNo1 polysaccharides	*Cordyceps militaris*	↓ TGF-β1, reduced fibrosis and ECM deposition	Yu et al. (79)

↑, increase/upregulation; ↓, decrease/downregulation.

A central mechanistic feature underlying these effects is the ability of mushroom-derived polysaccharides to modulate innate immune signaling and reprogram macrophage-driven inflammatory responses. In *Morchella esculenta*, polysaccharide administration significantly reduces circulating pro-inflammatory cytokines (IL-1β, TNF-α, IL-6) while simultaneously improving intestinal barrier integrity, suggesting a dual anti-inflammatory and gut-protective mechanism (58). This is particularly relevant in T2DM, where increased intestinal permeability and metabolic endotoxemia contribute to sustained systemic inflammation.

Similarly, *Inonotus obliquus*-derived low-molecular-weight polysaccharides attenuate inflammatory responses through suppression of NF-κB signaling and downregulation of TGF-β1-associated pathways, indicating broader regulatory effects that extend beyond classical inflammation into fibrotic and tissue-remodeling processes (77). Comparable NF-κB inhibitory effects have also been observed with polysaccharides from *Termitomyces albuminosus*, which reduce renal inflammatory burden in diabetic models (76).

In addition to cytokine suppression, mushroom bioactives also influence tissue-specific inflammatory and metabolic injury. For example, CmNo1 polysaccharides from *Cordyceps militaris* reduce TGF-β1 expression and extracellular matrix (ECM) deposition, thereby mitigating diabetic fibrosis and associated organ dysfunction (79). These findings highlight that immunometabolic regulation in T2DM is not limited to systemic cytokine control but also includes modulation of tissue remodeling and chronic injury pathways.

Importantly, these anti-inflammatory effects are tightly integrated with metabolic regulation. NF-κB signaling intersects with insulin receptor signaling pathways, and its chronic activation contributes directly to insulin resistance through inhibitory phosphorylation events and suppression of downstream metabolic signaling. Therefore, the observed reduction in inflammatory tone induced by mushroom-derived compounds likely contributes to improved insulin sensitivity by alleviating cytokine-mediated interference with insulin signaling networks.

Collectively, the available evidence supports the concept that medicinal mushrooms act as immunometabolic regulators, simultaneously targeting inflammatory signaling, tissue integrity, and metabolic homeostasis. This integrated effect reflects a systems-level mode of action in which immune modulation is not an isolated outcome but a core component of broader metabolic reprogramming in T2DM pathophysiology.

### 5.4 Gut microbiota as a central metabolic interface

The gut microbiota represents a key integrative interface linking dietary components, host metabolism, immune regulation, and systemic energy homeostasis. Across preclinical studies, mushroom-derived polysaccharides and associated bioactive compounds consistently modulate gut microbial composition and function, thereby influencing metabolic outcomes relevant to T2DM. These effects are mediated through microbiota-dependent production of short-chain fatty acids (SCFAs), regulation of bile acid metabolism, and reinforcement of intestinal barrier integrity, collectively contributing to improved glucose homeostasis and reduced metabolic inflammation (54, 82, 92, 98–124).

A central mechanistic axis in this context is the bile acid-gut microbiota-FXR/TGR5 signaling pathway. For instance, NAP-3 polysaccharide has been shown to regulate metabolic homeostasis through modulation of FXR/TGR5 signaling, indicating that fungal polysaccharides can indirectly influence host glucose and lipid metabolism via microbial-bile acid crosstalk (82). Similarly, polysaccharides from *Hericium coralloides* improve glycemic control through PI3K/Akt/GLUT4 signaling while simultaneously reshaping gut microbiota composition, highlighting a dual host–microbe mechanism of action (54).

In addition to metabolic signaling, several mushroom-derived compounds exert profound effects on intestinal ecosystem structure and barrier integrity. Polysaccharides from *Cordyceps militaris* not only remodel gut microbial communities but also enhance intestinal barrier function, with fecal microbiota transplantation (FMT) experiments confirming that metabolic benefits are at least partially microbiota-dependent (124). Likewise, *Grifola frondosa* polysaccharides ameliorate gut dysbiosis by promoting beneficial microbial phylotypes, reinforcing the role of dietary fungal glycans as prebiotic-like modulators of intestinal ecology (99).

Further supporting this concept, *Inonotus obliquus* polysaccharides improve intestinal barrier dysfunction, thereby reducing translocation of pro-inflammatory microbial products and attenuating metabolic endotoxemia, a key driver of chronic low-grade inflammation in T2DM (98). In parallel, betulinic acid derived from *Inonotus obliquus* increases the abundance of SCFA-producing bacteria, including *Lactobacillus* and *Eubacterium xylanophilum*, thereby enhancing production of metabolites that contribute to the regulation of insulin sensitivity, lipid metabolism, and gut-brain-endocrine signaling axes (92).

Collectively, these findings support the concept that mushroom-derived bioactive compounds act as microbiota-directed metabolic regulators, in which glycan structures function as fermentable substrates that reshape microbial ecology and metabolite output. The resulting increase in SCFA production, improvement in bile acid signaling, and reinforcement of intestinal barrier integrity converge to regulate systemic metabolic homeostasis.

From a systems biology perspective, the gut microbiota serves as a central mechanistic bridge connecting dietary mushroom intake to host metabolic regulation. Rather than requiring direct systemic absorption, many mushroom polysaccharides exert their effects indirectly through microbial fermentation and host–microbe metabolic crosstalk. This positions the gut microbiome as a critical mediator of the antidiabetic effects of medicinal mushrooms and highlights its importance in the development of functional-food-based strategies for T2DM prevention and management.

### 5.5 Molecular complexity, chemical synergy, and systems-level metabolic regulation

Medicinal mushrooms represent chemically and structurally complex biological systems in which bioactivity emerges from the coordinated interaction of multiple molecular classes operating across interconnected biological scales. Rather than acting as single-target agents, mushroom-derived constituents function as integrated metabolic modulators spanning extracellular glycan-mediated processes, intracellular small-molecule signaling, and host-microbiome communication networks.

A central feature of this system is the coexistence of high-molecular-weight polysaccharides and structurally diverse low-molecular-weight metabolites. Polysaccharides, particularly β-glucans and heteropolysaccharides, are primarily involved in modulation of gut microbiota composition, intestinal barrier integrity, and innate immune signaling. In contrast, low-molecular-weight metabolites, including triterpenoids (e.g., ganoderic acids), sterols, phenolic compounds, nucleoside analogs such as cordycepin, and sulfur-containing antioxidants such as ergothioneine act more directly on intracellular pathways governing metabolism, redox balance, and cellular energy sensing (101, 91).

Within this framework, biological effects arise from distributed pathway engagement rather than discrete ligand-target interactions. Polysaccharide-driven modulation of the gut microbiota alters short-chain fatty acid (SCFA) production and bile acid metabolism, thereby indirectly influencing host energy homeostasis, incretin secretion, and inflammatory tone. In parallel, small-molecule metabolites act at the level of intracellular signaling networks, including insulin-PI3K/Akt signaling, AMPK-mediated energy sensing, NF-κB-driven inflammatory pathways, and Nrf2-dependent redox regulation (102).

#### 5.5.1 Structural glycan organization as a functional determinant

A key determinant of this multi-level bioactivity is the structural plasticity of fungal glycans. β-glucans exhibit marked heterogeneity in glycosidic linkage patterns, branching architecture, molecular weight, and conformational behavior, including dynamic transitions between random-coil and triple-helical states. These supramolecular features are increasingly recognized as determinants of receptor recognition, immune activation, and microbial fermentability, although their precise contribution to *in vivo* metabolic outcomes remains incompletely defined (25, 26, 103). At the functional level, these determinants converge on three complementary routes to metabolic regulation: modulation of nutrient absorption kinetics, receptor-mediated immune signaling, and microbiota-dependent short-chain fatty acid production, thereby providing a mechanistic bridge between glycan architecture and downstream antidiabetic outcomes.

Emerging evidence further suggests that certain mushroom polysaccharides may interact with protein aggregation processes relevant to metabolic disease, including human islet amyloid polypeptide (hIAPP) involved in β-cell dysfunction in T2DM (45, 46). However, these findings are primarily derived from experimental systems, and their physiological relevance *in vivo* remains to be established.

Collectively, fungal glycans should therefore be viewed not merely as dietary fibers, but as structurally dynamic biomacromolecules capable of modulating immune, metabolic, and host–microbiome interfaces through both chemical composition and supramolecular organization.

#### 5.5.2 Chemical diversity and interaction-based bioactivity

In parallel, the low-molecular-weight metabolite fraction contributes a chemically diverse bioactive repertoire with partially complementary functions. Phenolic compounds and ergothioneine provide redox buffering and mitochondrial protection, triterpenoids and sterols may regulate lipid metabolism and intracellular signaling, while cordycepin modulates energy metabolism and SIRT-associated pathways (101, 91).

Rather than acting independently, these compounds may generate context-dependent interaction effects, resulting in additive or partially synergistic biological outcomes. However, current evidence remains insufficient to define the nature, consistency, or magnitude of such interactions, largely due to reliance on isolated-compound studies or complex extracts that preclude mechanistic resolution.

#### 5.5.3 Systems-level integration and network pharmacology perspective

At the systems level, these multi-component effects converge on a limited set of core regulatory axes central to T2DM pathophysiology, including insulin signaling, AMPK-mediated energy sensing, redox homeostasis, inflammatory signaling, and gut microbiota-host metabolic crosstalk. In this context, medicinal mushrooms can be conceptualized as network-active biological systems rather than collections of independent bioactive molecules.

This interpretation aligns with a network pharmacology framework, in which therapeutic effects emerge from simultaneous, moderate modulation of multiple interconnected nodes rather than high-affinity interaction with single targets. Such a mechanism is particularly relevant for T2DM, a disorder characterized by progressive failure of integrated metabolic networks rather than a single dominant molecular defect.

However, mechanistic evidence remains largely correlative. Most studies rely on pathway expression or phosphorylation changes, while causal validation using genetic, pharmacological, or microbiome-transfer approaches remains limited. Future work should therefore aim to establish causal hierarchies within these networks and distinguish direct molecular effects from host–microbiome-mediated indirect mechanisms.

From a translational perspective, this complexity presents both opportunities and constraints. Whole mushroom extracts may preserve emergent multi-component interactions contributing to efficacy, whereas purified compounds provide mechanistic precision but may not fully recapitulate system-level behavior. Resolving this balance will be essential for the rational development of standardized, evidence-based mushroom-derived functional foods and nutraceutical interventions.

## 6 Translational relevance, limitations, and caveats

Despite growing interest in medicinal mushrooms as functional foods or nutraceutical candidates for T2DM, the current evidence base remains insufficient to support strong clinical conclusions. Most available data derive from preclinical studies, often using chemically induced or genetic diabetic models, relatively high doses of extracts, and short intervention periods (58–60, 66–54). While these studies provide useful biological hypotheses, they do not reliably establish clinical efficacy, preventive benefit, or therapeutic utility in humans. Translation into clinical practice is therefore limited by major methodological, mechanistic, and practical constraints.

A central limitation is that much of the mechanistic evidence remains associative rather than causally validated. Many studies report changes in phosphorylation status, protein abundance, gene expression, antioxidant enzyme activity, cytokine levels, lipid markers, or gut microbiota composition after treatment with mushroom-derived extracts or isolated compounds (59, 60, 67–71, 76, 54–84, 86–89, 91–95, 97–100, 104). These readouts suggest possible involvement of pathways such as PI3K/Akt, AMPK, Nrf2/HO-1, NF-κB, SIRT-dependent signaling, FXR/TGR5, and GLP-1-related mechanisms, but they do not necessarily demonstrate that these pathways are required for the observed metabolic effects. In many cases, functional validation using pharmacological inhibitors, genetic knockdown, receptor blockade, glucose uptake assays, kinase activity measurements, metabolic flux analysis, germ-free or antibiotic-treated models, or fecal microbiota-transfer experiments is absent. Consequently, many proposed mechanisms should be interpreted as pathway-associated or hypothesis-generating rather than pathway-validated.

The clinical evidence is even more limited. Human studies in patients with established T2DM are scarce, generally short in duration, and heterogeneous in study design, participant characteristics, background medication use, mushroom species, intervention format, dose, and outcome measures (62–64). Most trials focus primarily on glycemic and lipid parameters, while mechanistically informative endpoints such as oxidative stress biomarkers, inflammatory mediators, gut microbiota composition, bile acid metabolism, incretin responses, and pharmacokinetic profiles are inconsistently assessed or absent (61–64). This creates a substantial gap between the mechanistic claims derived from preclinical models and the endpoints measured in clinical studies. At present, the available clinical literature should be viewed as preliminary and exploratory rather than confirmatory.

Another major barrier is the poor standardization of mushroom-derived interventions. Preparations differ substantially with respect to species, strain, cultivation substrate, developmental stage, fruiting body versus mycelium, extraction solvent, extraction temperature, purification method, storage conditions, molecular-weight distribution, β-glucan content, triterpenoid profile, phenolic composition, ergothioneine content, cordycepin content, and other bioactive constituents (20, 21, 25–27, 34, 47–51, 56, 57, 105). In many studies, the chemical composition of the intervention is incompletely characterized, and species authentication is not always clearly reported. Without molecular identification, voucher specimens, batch-to-batch compositional analysis, and quantitative profiling of major bioactive classes, reproducibility remains weak and cross-study comparison is difficult. This limitation is particularly important because two products labeled with the same mushroom species may differ substantially in biological activity.

Dose-response relationships also remain poorly defined. Preclinical studies often use doses that may not correspond to realistic dietary exposure in humans, while clinical studies vary widely in dose, duration, and preparation type (58–64, 80–89). It remains unclear whether regular consumption of edible mushrooms can produce clinically meaningful metabolic effects, or whether concentrated and chemically standardized extracts are required. This distinction is important because whole-food interventions may offer better dietary feasibility and safety, whereas concentrated extracts may produce higher pharmacological exposure but also require more rigorous evaluation of pharmacokinetics, toxicity, and interactions with standard antidiabetic therapies (20, 27, 105). Without dose-finding studies and human exposure data, it is difficult to define biologically relevant or clinically appropriate intake levels.

Pharmacokinetic and bioavailability issues represent an additional unresolved problem. High-molecular-weight polysaccharides are unlikely to be absorbed intact to a meaningful extent and may act primarily through intestinal, immune, or microbiota-mediated mechanisms, including modulation of microbial fermentation, short-chain fatty acid production, bile acid signaling, and intestinal barrier function (33, 54, 82, 86, 92, 98–100, 103, 104). In contrast, lower-molecular-weight compounds such as triterpenoids, phenolics, ergothioneine, and cordycepin may undergo absorption, metabolism, tissue distribution, and clearance, but these processes remain incompletely characterized in humans (47–101, 52, 91). For example, cordycepin has major translational limitations because it is rapidly metabolized by adenosine deaminase and cleared quickly, motivating strategies such as ADA inhibitors, derivatives, or delivery systems (106). Poor solubility, metabolic instability, transporter dependence, first-pass metabolism, and rapid elimination may all limit systemic exposure. Therefore, *in vitro* activity or efficacy in animal models cannot be assumed to translate into clinically relevant effects without pharmacokinetic and metabolomic confirmation.

Safety also requires more critical consideration. Edible mushrooms are generally consumed safely as part of the diet, but concentrated extracts and nutraceutical formulations may produce different exposure profiles (20, 26, 27, 105). Potential concerns include additive hypoglycemic effects when combined with antidiabetic drugs, additive hypotensive effects with antihypertensive agents, and possible bleeding risk when used with anticoagulant or antiplatelet therapy (107–109). Rare reports of liver injury associated with *Ganoderma*-containing products indicate that hepatotoxicity, although uncommon, should not be dismissed, particularly for concentrated or poorly characterized preparations (110–113). In addition, mushrooms can bioaccumulate potentially toxic elements such as cadmium (Cd), lead (Pb), mercury (Hg), and arsenic (As) from soil or cultivation substrates, depending on species, substrate, cultivation conditions, and geographic origin (114–118). This is directly relevant to several species discussed in this review, including *Ganoderma lucidum*, *Agaricus bisporus*/blazei, *Pleurotus ostreatus*, *Lentinula edodes*, *Cordyceps*/*Ophiocordyceps sinensis*, *Trametes versicolor*, *Phellinus linteus*, and *Poria/Wolfiporia cocos*. Contaminant screening should therefore be considered essential for products intended for clinical or long-term nutritional use.

Overall, the current literature supports biological plausibility but not definitive clinical efficacy. As discussed in Section “4.2 Clinical evidence,” the only placebo-controlled RCT in patients with T2DM/metabolic syndrome found no significant effect on glycemic or cardiovascular risk markers, underscoring this gap (65). Beyond studies directly performed in patients with T2DM, recent randomized clinical evidence from metabolically at-risk populations provides additional insight into the cardiometabolic effects of mushroom-derived compounds. A double-blind placebo-controlled trial investigated *Pleurotus ostreatus* powder enriched in fungal β-glucans (8.4 g/day providing 3 g β-glucans) in adults with moderately elevated LDL cholesterol. Although supplementation for 4 weeks did not significantly alter LDL-C, total cholesterol, triglycerides, or apolipoprotein concentrations, exploratory analyses suggested modulation of cholesterol absorption markers, particularly in females. These findings indicate that mushroom-derived β-glucans may influence specific metabolic pathways; however, the absence of clinically meaningful lipid improvements highlights the need for longer interventions and studies performed specifically in diabetic populations (119). Medicinal mushrooms and their bioactives should therefore be regarded as promising but insufficiently standardized candidates for further investigation, rather than established interventions for T2DM prevention or treatment. Future studies must move beyond descriptive biomarker changes and adopt more rigorous experimental and clinical designs. Priorities include standardized species authentication, quantitative compositional profiling, dose-response studies, pharmacokinetic assessment, safety monitoring, evaluation of mushroom–drug interactions, and adequately powered long-term clinical trials. Such studies should integrate conventional metabolic outcomes with mechanistic endpoints, including redox balance, inflammatory status, gut microbiota dynamics, bile acid signaling, incretin responses, and validated pathway-specific assays (18, 20, 27, 61–64, 103, 105–107). Only through this more rigorous framework can the true translational relevance of medicinal mushrooms in T2DM be determined.

## 7 Perspective and future directions

Future research should prioritize well-designed, adequately powered, long-term clinical trials in individuals with prediabetes, insulin resistance, metabolic syndrome, or established T2DM (18, 27). These studies should employ standardized mushroom preparations with clearly defined doses and well-characterized bioactive profiles. Clinically relevant endpoints should include fasting glucose, HbA1c, postprandial glucose, HOMA-IR, lipid profile, body composition, inflammatory markers, oxidative stress biomarkers, gut microbiota composition, GLP-1 signaling, and liver fat indices. Importantly, future trials should clearly distinguish between dietary mushroom intake, whole-powder preparations, polysaccharide-rich extracts, mycelial biomass, fruiting-body preparations, and purified bioactive compounds, as cultivation conditions, developmental stage, extraction methodology, and processing techniques can substantially alter secondary metabolite composition and biological activity (105).

A critical priority is the standardization of extraction, characterization, and reporting protocols to ensure reproducibility and cross-study comparability. Variability in species, strain, substrate, cultivation conditions, extraction solvents, molecular-weight distribution, β-glucan content, triterpenoid profiles, phenolic composition, and storage conditions currently represents a major barrier to translational progress. The development of standardized functional food formulations may facilitate broader clinical application of mushroom-derived interventions as adjunct nutritional strategies; however, this requires rigorous compositional profiling, dose optimization, safety evaluation, and long-term efficacy assessment in relevant metabolic populations (120).

From a mechanistic standpoint, future studies should integrate multi-omics approaches, including metabolomics, glycomics, microbiome profiling, and transcriptomics, combined with pathway-specific validation. In particular, it remains essential to determine whether key signaling pathways such as PI3K/Akt, AMPK, Nrf2, NF-κB, SIRT3, FXR/TGR5, and GLP-1 are causally required for the metabolic effects of mushroom bioactives. Causal inference approaches, including pharmacological inhibition, genetic knockdown, germ-free and antibiotic-treated models, and microbiota-transfer experiments, will be essential to distinguish direct effects of fungal compounds from microbiota-mediated mechanisms (121). Additionally, integration of ethnopharmacological knowledge with modern mechanistic platforms may help identify underexplored species, including *Pleurotus florida* and termite-associated mushrooms, which have shown preliminary metabolic activity (122).

From a chemical and structural perspective, deeper investigation of fungal glycans and low-molecular-weight metabolites is warranted. Key structural determinants of polysaccharide bioactivity include glycosidic linkage patterns, branching architecture, molecular weight distribution, solubility, conformational dynamics, and supramolecular organization. Understanding how these structural features influence receptor binding, fermentability, and immune-metabolic signaling will be crucial for rational development. In parallel, systematic evaluation of interactions between polysaccharides and small molecules such as ganoderic acids, ergothioneine derivatives, cordycepin analogs, and phenolic compounds is needed to clarify potential synergistic or context-dependent effects. Advances in synthetic chemistry, semi-synthesis, and extraction standardization will further enable structure–activity relationship studies and functional optimization.

Collectively, these integrated approaches will be essential for transitioning medicinal mushrooms from empirically used functional foods into evidence-based, mechanistically defined nutritional or therapeutic strategies for the prevention and management of T2DM.

## 8 Conclusion

Medicinal mushrooms represent a highly complex and multifunctional source of bioactive compounds capable of modulating interconnected biological pathways involved in the pathogenesis of T2DM, including insulin signaling, oxidative stress, inflammatory responses, mitochondrial function, and gut microbiota-host interactions. Current evidence suggests that a broad spectrum of fungal constituents, including polysaccharides, β-glucans, triterpenoids, phenolics, sulfur-containing metabolites, and other bioactive molecules, collectively contribute to improved metabolic resilience and cardiometabolic health through coordinated multi-target effects.

Although preclinical and mechanistic findings are encouraging, clinical evidence remains limited, and significant challenges related to standardization, bioavailability, mechanistic causality, and long-term translational validation remain unresolved. Addressing these limitations will require integrative research approaches combining glycomics, metabolomics, microbiome profiling, supramolecular structural characterization, and rigorously designed clinical trials. Therefore, medicinal mushroom-derived products should currently be viewed as promising but insufficiently standardized functional-food candidates, rather than clinically validated interventions for T2DM prevention or treatment.

Collectively, medicinal mushrooms should be considered not merely as nutritional supplements, but as complex biological systems with the potential to inform the development of evidence-based functional food strategies for the prevention and management of T2DM and related metabolic disorders.
